# Broadband Bending of Flexural Waves: Acoustic Shapes and Patterns

**DOI:** 10.1038/s41598-018-29192-1

**Published:** 2018-07-25

**Authors:** Amir Darabi, Ahmad Zareei, Mohammad-Reza Alam, Michael J. Leamy

**Affiliations:** 1 School of Mechanical Engineering, Georgia Institute of Technology, Atlanta, 30332 USA; 2 Mechanical Engineering Department, University of California, Berkeley, 94703 USA

## Abstract

Directing and controlling flexural waves in thin plates along a curved trajectory over a broad frequency range is a significant challenge that has various applications in imaging, cloaking, wave focusing, and wireless power transfer circumventing obstacles. To date, all studies appeared controlling elastic waves in structures using periodic arrays of inclusions where these structures are narrowband either because scattering is efficient over a small frequency range, or the arrangements exploit Bragg scattering bandgaps, which themselves are narrowband. Here, we design and experimentally test a wave-bending structure in a thin plate by smoothly varying the plate’s rigidity (and thus its phase velocity). The proposed structures are (i) broadband, since the approach is frequency-independent and does not require bandgaps, and (ii) capable of bending elastic waves along convex trajectories with an arbitrary curvature.

## Introduction

While acoustic waves propagate along a straight line in a homogeneous medium, metamaterials are capable of diffracting and directing acoustic waves by spatially varying the host material properties along the wave propagation, and have various applications such as health monitoring^1,2^, telecommunication^3^, super-resolution acoustic imaging in surgery^4–6^, cloaking^7–12^, wave focusing^13–16^, wave guides^17–21^, and wave bending for electronic devices^1,22^.

Current approaches for manipulating and bending flexural waves depend on either changing the effective refractive index to steer waves^14,23,24^, or exploiting frequency bandgaps to guide waves along a pre-defined path^21,25–28^. Both approaches can be achieved using periodic structures such as phononic crystals and metamaterials^29^. These periodic structures are lattice materials exhibiting refractive frequency bandgaps that can be tailored via unit cell design. A few recent studies report efforts to bend or steer acoustic/elastic waves without using phononic crystals or metamaterials. Zhang *et al*. introduced the idea of three-dimensional acoustic bottles generated by self-bending beams in a homogeneous acoustic medium. Phase changes in a linear array of 40 sources produce the necessary self-bending, and thus acoustic energy can be propagated along curved paths which circumvent obstacles^30^. Tol *et al*. implemented a two-dimensional version of such systems for flexural waves in thin plates using sources composed of piezoelectric transducers^31^. The acoustic bottle approach is broadband, to arbitrary frequency, as the number of sources in the phased array approaches infinity (since the source spacing must be less than a wavelength). Note that for most of applications in wave bending devices (e.g. health monitoring, focusing, or wave-guiding/wave-bending devices) the incoming wave is given, and engineering the initial wave packet is not feasible.

The primary aim of this Letter is to propose an alternative approach for bending flexural waves along any curved trajectories in thin plates by machining the surface of the host medium along a desired path. This new technique is broadband for the lowest asymmetric Lamb wave, and works for any convex trajectory without introducing a symmetric artifact. The wave controlling structure is created by continuously altering the flexural rigidity profile (i.e. plate’s thickness) similar to that used previously to design a flexural continuous gradient-index (GRIN) lens concept^16^. A model capturing the essential ideas of the system is introduced first, followed by numerical results generated using Finite Element Methods. Moreover, the tailored system is tested experimentally to demonstrate the bending of elastic waves over a broad frequency range (20 kHz–120 kHz), which is limited by the frequency bounds of the experimental setup.

## Results

### Modeling

The governing equation of flexural waves in a thin plate with elasticity modulus *E*, thickness *h*, density *ρ*, and Poisson’s ratio *ν* is given by

1 $$D{\nabla }^{4}w+\rho h{w}_{tt}=\mathrm{0,}$$

where $D=E{h}^{3}/12(1-{\nu }^{2})$ represents the flexural rigidity, *w* the transverse displacement, and subscript *tt* denotes a second derivative with respect to time. Equation (1) is known as Kirchhoff-Love plate equation and is valid when the wavelength *λ* is large enough compared to the thickness of the plate *h* and small compared to it’s in-plane dimension *L* (i.e. $h\ll \lambda \ll L$). The dispersion relation of plane flexural waves with wavenumber *k* is then obtained as $D{k}^{4}-\rho h{\omega }^{2}=0$, where the phase velocity is

2 $${v}_{p}^{4}={(\frac{\omega }{k})}^{4}={\omega }^{2}\frac{E{h}^{2}}{\mathrm{12(1}-{\nu }^{2})\rho }\mathrm{.}$$

**Figure 1:**
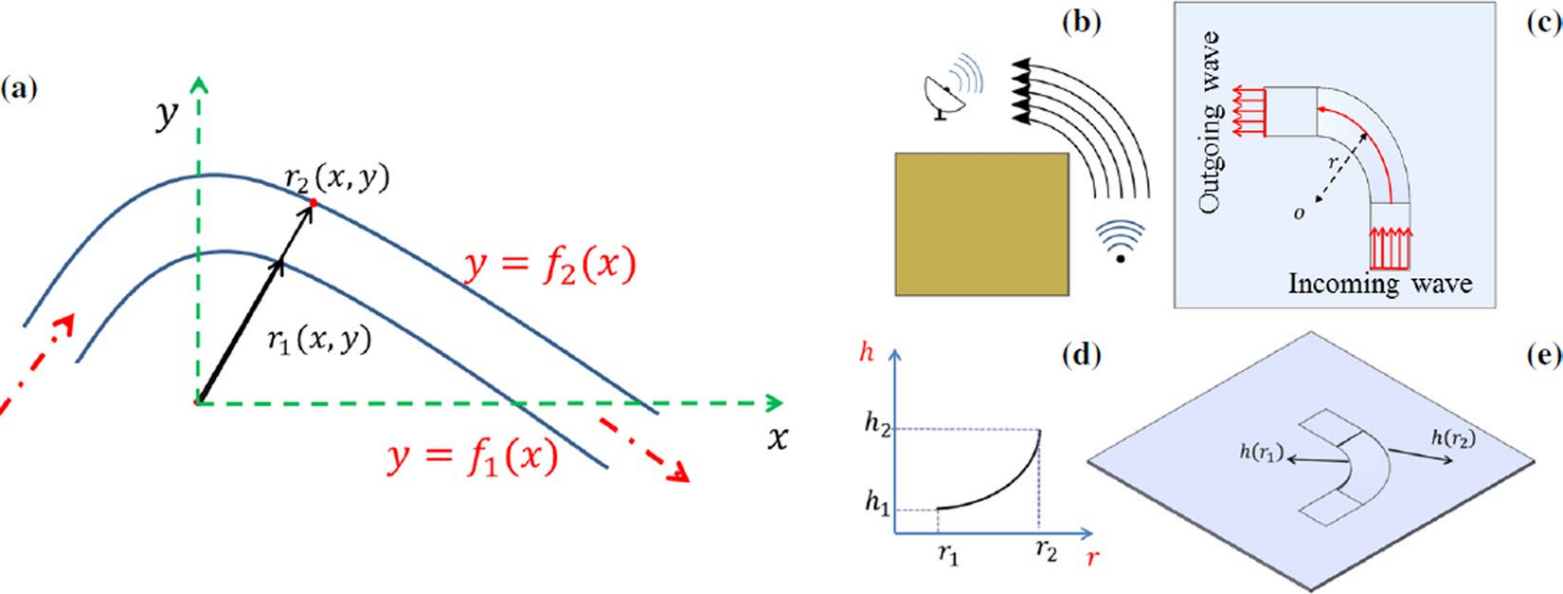
Overview of the proposed wave-bending approach. **(a)** Geometry associated with wave bending on a flat plate. **(b)** Schematic of the proposed structure to bend a flexural wave from a source to a receiver. **(c)** Top view of the structure serving as the host medium and the machined channel to bend the wave. **(d)** Thickness profile of the channel; and **(e)** Schematic of the thickness variation along the guiding trajectory.

We assume a channel with a convex trajectory for bending the traveling wave (Fig. 1). As shown, the channel is bounded by two desired curves *y* = *f*_1_(*x*) and *y* = *f*_2_(*x*) with the same center of curvature and curvature radii *r*_1_(*x*, *y*) and *r*_2_(*x*, *y*) respectively. The radius of each curve is obtained as^32^ $r(x,y)={(1+{y}_{x}^{2})}^{\frac{3}{2}}/|{y}_{xx}|$, where subscript *x* denotes a derivative with respect to *x*. In order to design a bending acoustic device, the phase fronts need to rotate around the center of curvature with a constant angular frequency. Therefore, the phase velocity along the channel should vary as ${v}_{p}(r)=r{\bar{\omega }}_{p}$, where ${\bar{\omega }}_{p}$ is the angular speed associated with wave bending. Considering the relation between the phase velocity and the thickness of the plate in Eq. (2), and the equation for phase velocity $({v}_{p}(r)=r{\bar{\omega }}_{p})$, wave bending in thin plates is achievable if the thickness varies continuously as,

3 $$h=\frac{{(r{\bar{\omega }}_{p})}^{2}}{\omega }\sqrt{\frac{\mathrm{12(1}-{\nu }^{2})\rho }{E}}\mathrm{.}$$

This implies $h\propto {r}^{2}$ and requires the plate’s thickness to vary quadratically between the inner radius of the created channel *h*_1_ = *h*(*r*_1_), and the outer radius *h*_2_ = *h*(*r*_2_) (Fig. 1b–e). Note that different values of inner and outer thickness for the quadratic thickness profile would result in different angular frequency for the rotational speed of phase fronts.

### Numerical results

**Figure 2:**
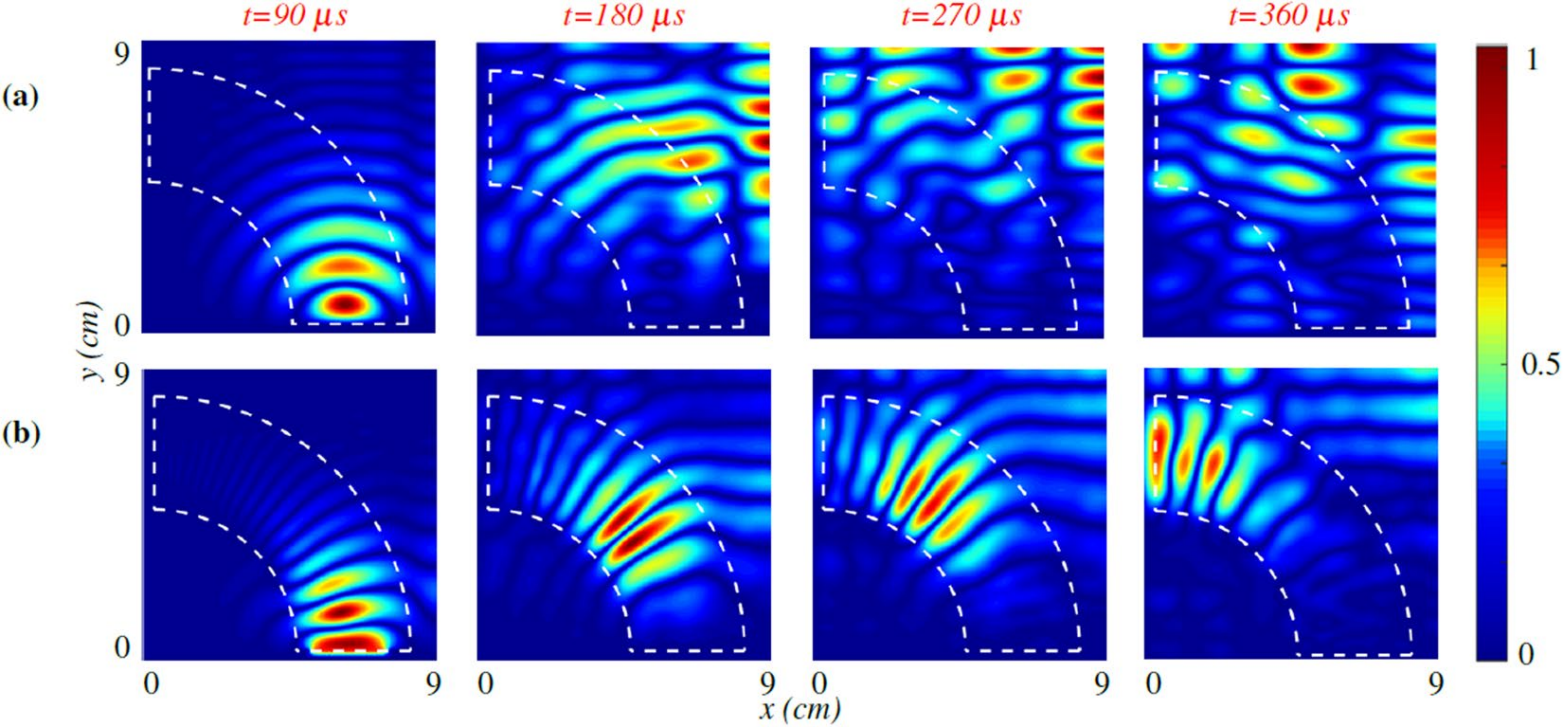
Numerical results. Numerically calculated wave response of the system at the frequency of 50 *kHz*. Waveguide’s boundary is shown with white dashed line where the boundaries are at *r*_1_ = 5 *cm* and *r*_2_ = 8.6 *cm*. **(a)** Constant thickness h = 1 *mm*, along the guiding trajectory different from host’s medium thickness. **(b)** Proposed thickness *h*(*r*) = 0.4 *r*^2^ along the wave bending channel.

Numerical simulations using the Finite Element Methods (FEM) are first employed to exhibit the effectiveness of the proposed wave bending structure. To support the importance of thickness variation in bending waves, two different cases have been considered. For both cases, Aluminum plate with a constant thickness, *h*_0_ = 3.175 *mm* serves as the host medium of the waveguide channel with the inner and outer radius of ${r}_{1}=5\,cm,{r}_{2}=8.6\,cm$. (see Methods for details). For the first case, we assume a constant thickness (*h*(*r*) = 1 *mm*) in the channel, while for the second case the quadratic thickness profile follows *h*(*r*) = 0.4 *r*^2^. Accordingly, for the latter case, the thickness varies with a quadratic profile between *h*_1_ = 1 *mm* and *h*_2_ = 3 *mm* in the channel. Fig. 2 depicts the computed time snapshots of wavefield for both cases at 50 *kHz* (the first row of subfigures depicts the results for the constant thickness waveguide, and the second row shows the results for the quadratic thickness profile). Note that the results have been normalized with respect to the amplitude of incoming plane wave. Figure 2a confirms that the impedance mismatch at the waveguide’s boundary is not sufficient for confining the waves inside the channel and bending the waves in the curved trajectory waveguide. As observed in Fig. 2b, the proposed wave bending structure with quadratic thickness profile, bends the traveling wave fronts and is capable of confining the flexural waves inside the waveguide. It is to be noted that the leakage from the waveguide is minimal and thus the wave intensity decreases slightly as it bends along the desired path.

### Experimental results

**Figure 3:**
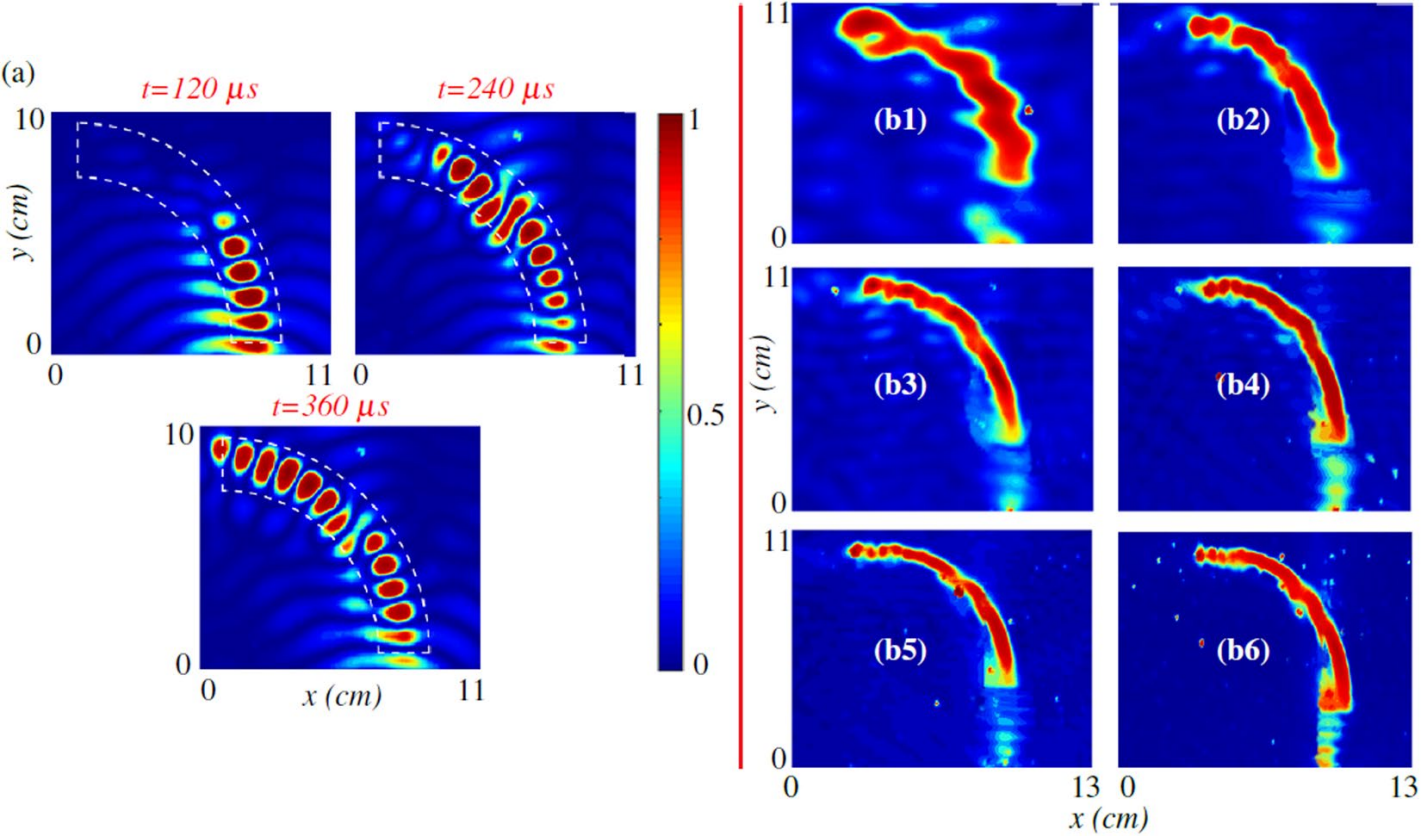
Experimental results. (**a)** Experimentally measured wave response of the proposed wave-bending structure at 50 *kHz*, Experimentally measured nondimensionalized wavefield displacement generated by line source excitation at **(b1)** *f* = 20 *kHz*, **(b2)** *f* = 40 *kHz*, **(b3)** *f* = 60 *kHz*, **(b4)** *f* = 80 *kHz*, **(b5)** *f* = 100 *kHz*, and **(b6)** *f* = 120 *kHz*.

Next, a set of experiments is carried-out to verify the performance of the proposed wave-directing structure (see Methods for details). Fig. 3a provides snapshots of the experimentally-measured wavefield displacements in response to excitation at 50 *kHz* for the waveguide discussed in the numerical section. Note that the results have been normalized with respect to the amplitude of the wave near the source. In qualitative agreement with the numerical simulations, the traveling wave propagates along the trajectory with a low amplitude loss and wave leakage outside the channel. Figure 3b depicts the measured RMS wavefield obtained experimentally at six different frequencies. These figures confirm the wavefront bending in the proposed waveguide over a broad range of frequencies (i.e. 20–120 *kHz*). The weak leakage of wave energy outside the channel is due to the discrete nature of maufacturing process and the as-manufactured profile.

**Figure 4:**
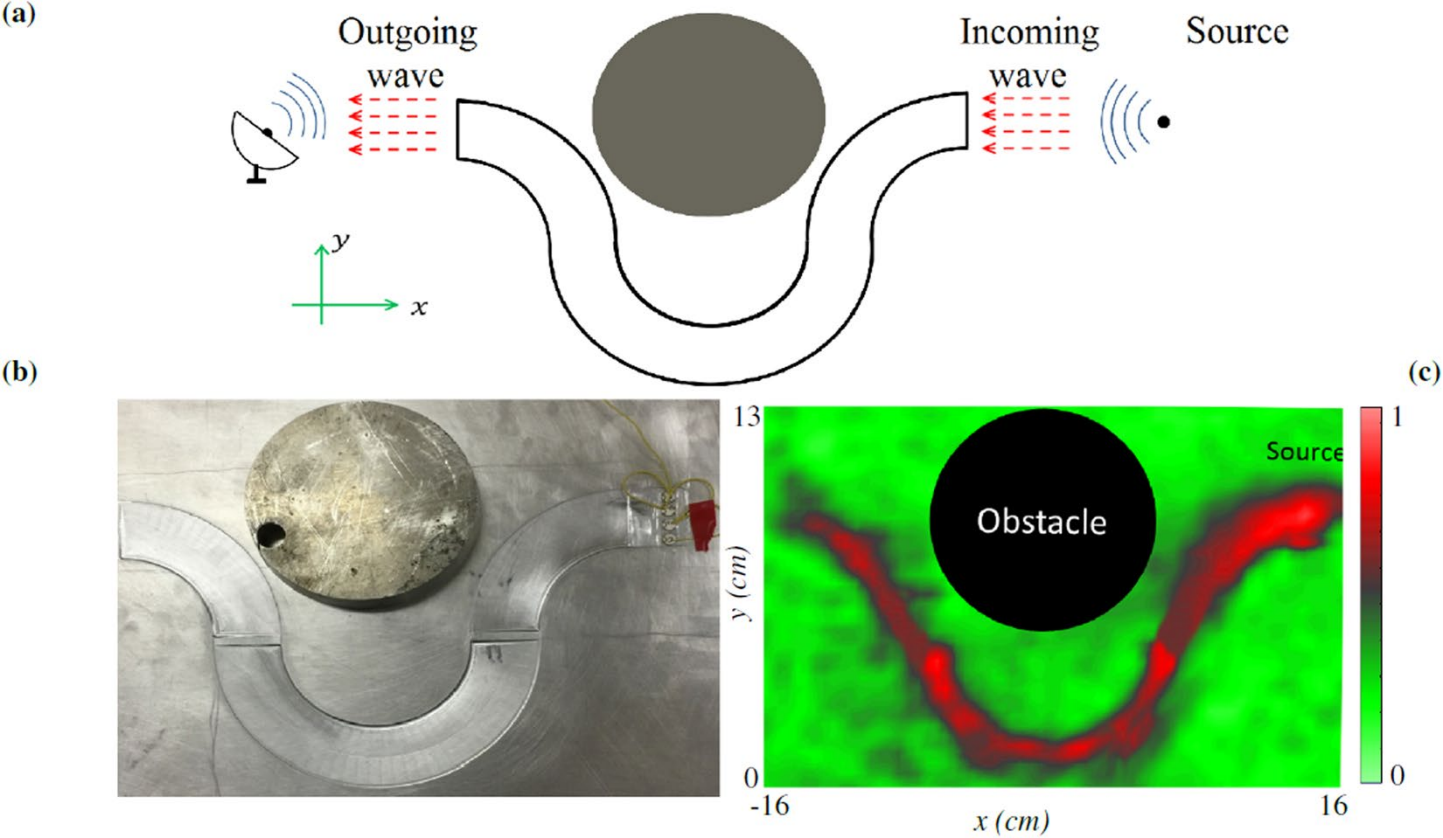
Experimentally measured wave circumventing around an obstacle. (**a**) Schematic of the proposed method to rotate the traveling wave around an obstacle, **(b)** experimental modeling, and **(c)** normalized RMS wavefield response of the system to a harmonic incoming plane wave at the frequency of 50 *kHz*.

Navigating waves around an obstacle is one potential use for the proposed wave-bending structures. To support this application, another experimental setup (Fig. 4a) is designed and tested (see Methods). A thin aluminum plate with a width of *w* = 25 cm, length of *L* = 28 cm, and thickness of *h*_0_ = 3.175 *mm* again serves as the host medium (Fig. 4b). The wave channel is formed by connecting three quarter-circles with inner and outer radii of *r*_1_ = 5 *cm*, *r*_2_ = 9 *cm*, respectively. The thickness of the channel varies from *h*_1_ = 1 *mm* at *r*_1_ to *h*_2_ = 3 *mm* at *r*_2_ with a parabolic profile to satisfy equation (3). Note that, since abrupt thickness variation is not desired when the curvature changes, a transient region has been made on the plate with the thickness of 3 *mm*, and the length of 3 *mm*. A steel cylinder with a radius of 7.5 *cm* and tickness of 2 *cm* is attached to the host plate to replicate an obstacle. Four epoxy-bonded piezoelectric transducers with thickness and diameter of ${h}_{p}=0.4\,mm,\,{d}_{p}=5$ *mm* (Steiner Martins SMD05T04R411, 3 M DP270 Epoxy Adhesive) located 2 *cm* away from the leading edge of the channel produce an incident wave in response to a generated 200 *mV* (peak-to-peak) voltage profile using 10 sinusoidal cycles. The experimentally-measured RMS wavefield at *f* = 50 *kHz* is depicted in Fig. 4c. This subfigure clearly documents the desired bending of the wave around the obstacle. As observed, the wave amplitude is re-amplified at the points where the curvature changes. One possible explanation is that the amplified region is experiencing near-resonance behavior due to reflections at its boundaries. Some loss of wave intensity is noted, which can be expected due to material losses associated with the increased propagation distance (≈50 *cm*) or weak leakage of propagating wave outside the channel.

**Figure 5:**
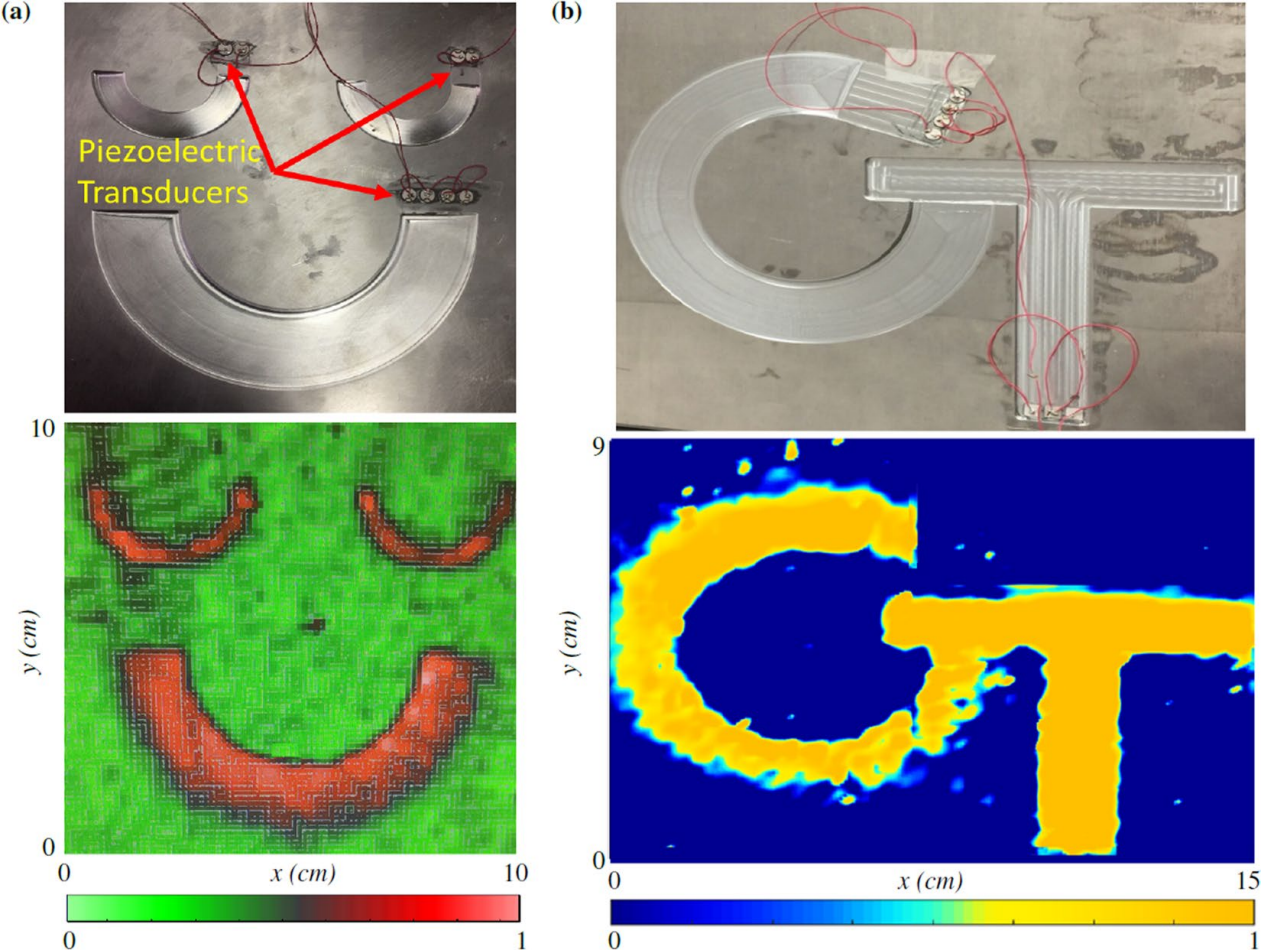
Experimentally measured patterns and shapes. Experimentally-measured normalized RMS wave response generated by piezoelectric transducers at *f* = 50 *kHz* for **(a)** a smiley face, **(b)** GT logo.

In other applications, wave bending can be employed to create acoustic patterns and shapes, which themselves may discreetly encode information (i.e. the information would only be available to those who know to look for it). Figure 5a depicts an engineered surface leading to a semi-circles path to form a “smiley” face inspired, in part, by similar smiley patterns created using folded DNA^33^. Note that six piezoelectric transducers (SMD063T07R111) are used to excite the system (see Methods). Due to the shape of the structure in Fig. 5, propagating waves in the opposite direction could destruct the wave-field in the smaller curves. To overcome this issue, wave propagation is prevented in the opposite direction by applying absorbing pitch tapes to the surface of the plate behind the piezoelectric transducers. The RMS wave response of this pattern is shown in Fig. 5b at 50 *kHz*. In a final example, a Georgia Tech (GT-shaped) logo is created and tested at 50 *kHz*. Figure 5b details the engineered surface and the resulting RMS wavefield of the structure. These figures clearly represent the power of the introduced technique to bend and direct flexural waves along desired trajectories, resulting in novel acoustic shapes and patterns.

## Conclusion

In summary, this letter proposed a novel, broadband approach for bending flexural waves along convex trajectories by tailoring the medium’s flexural rigidity. The configured structures are capable of guiding and bending plane waves for subsequent use in wave manipulation, obstacle avoidance, wireless power transfer, and information encoding. We numerically explored the design and performance of the structures, and verified the approach using experimental measurements. With further development, the proposed approach may find application, for example, in vehicle unibodies to channel noise paths for subsequent absorption, or in seismic protection where tailored terrain will bend surface waves away from susceptible structures.

## Methods

### Numerical modeling

The Finite Element Method (FEM) is used to solve the governing equation (1) subject to a desired thickness variation^16^. The numerical domain is set to [0, 9] *cm* × [0, 9] *cm* for the host medium with a thickness of *h*_0_ = 3.175 *mm* (Fig. 1c). The wave bending channel is formed in the host layer between two quarter of a cricle with radii *r*_1_ = 5 *cm*, and *r*_2_ = 8.6 *cm*. The plate’s thickness varies with a parabolic profile *h*(*r*) = 0.4 *r*^2^ where the inner and outer thickness in the waveguide are *h*_1_ = 1 *mm*, and *h*_2_ = 3 *mm* respectively (Fig. 1d). The numerical domain is seeded using nodes spaced by Δ*x* = 0.15 *mm* and element mesh is created using the FreeFEM++ ^34^ mesh generator. Perfectly matched layer (PML) boundary conditions are used to absorb outgoing waves at the plate’s edges. A single sine wave with the wavelength and speed matching with excitation frequency is located at the start of the channel to initialize the wavefield as a source.

### Experimental measuring

**Figure 6:**
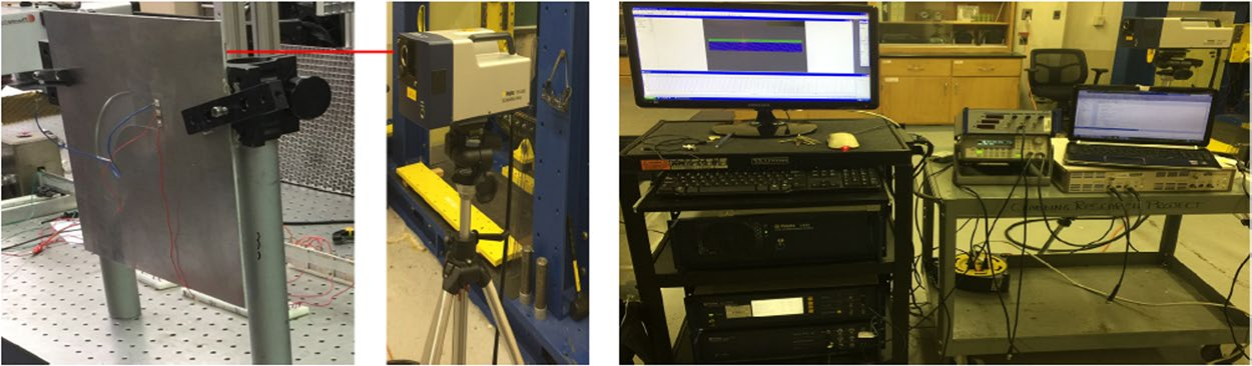
Experimental setup showing an aluminum plate hosting piezoelectric transducers for exciting waves, a function generator and amplifier for generating requisite voltage profiles, and a laser vibrometer for measuring the backside transverse plate velocities.

As depicted in Fig. 6, a Polytec PSV-400 scanning laser Doppler vibrometer measures the resulting out-of-plane wavefield velocity using the backside of a thin aluminum plate. The plate chosen has a width of *w* = 12 *cm*, length of *L* = 14 *cm*, and thickness of *h*_0_ = 3.175 *mm*. The wave-guide channel is created by quadratic thickness profile varying between *h*_1_ = 1 *mm* at *r*_1_ = 5 *cm* to *h*_2_ = 3 *mm* at *r*_2_ = 8.6 *cm*. The thickness machining was performed at the Georgia Tech Montgomery Machining Mall using a CNC mill. The wavefileld displacement is scanned over a 11 *cm* × 13 *cm* square area with a 250 × 250 grid resolution and a time resolution of *δt* = 1 *μ*s. Absorbing pitch tape is used to mitigate reflecting wave at the boundaries. Three *h*_*p*_ = 0.2 *mm* thick epoxy-bonded piezoelectric transducers (Steiner Martins SMPL30W30T1121, 3M DP270 Epoxy Adhesive) located 1 *cm* away from the channel produce an incident wave in response to a generated 200 *mV* (peak-to-peak) voltage profile by 5 cycles of sinusoidal wave (10 cycles in the obstacle experiment), using a function generator (Agilent 33220A) coupled to a voltage amplifier (B&K1040L). Each of these piezoelectric disks provides an in-plane vibration perpendicular to the surface of this piezoelectric acting as a point source. Due to the operating frequency range of the system, having an appropriate number of these disks (depends on the length of the incident wave) will form a wave close to a harmonic plane wave. Note that to make the wave propagate the least outside the channel, a rectangular cavity has been made where transducers have been placed (a layer with the thickness of 3.1 *mm*). Each of these piezoelectric transducers measure 7 mm × 7 mm and have an effective capacitance of *C*_*p*_ = 1.5 *nF*. Proper triggering of the laser measurements allows the reconstruction of the out-of-plane velocity field while the RMS (root-mean-square) values are obtained by integrating the measured response over time.
